# Prognostic Value of Perineural Invasion in Esophageal and Esophagogastric Junction Carcinoma: A Meta-Analysis

**DOI:** 10.1155/2016/7340180

**Published:** 2016-03-08

**Authors:** Aiqin Gao, Linlin Wang, Juan Li, Hongyu Li, Yali Han, Xiaoxia Ma, Yuping Sun

**Affiliations:** ^1^Department of Oncology, Jinan Central Hospital Affiliated to Shandong University, Jinan 250013, China; ^2^Department of Radiotherapy, Shandong Cancer Hospital, Jinan 250117, China; ^3^School of Medicine, Shandong University, Jinan 250012, China; ^4^Department of Medical Insurance, Jinan Central Hospital Affiliated to Shandong University, Jinan 250013, China; ^5^Endoscopy Center, Jinan Central Hospital Affiliated to Shandong University, Jinan 250013, China

## Abstract

*Objective*. Here we aimed to clarify the prognostic significance of perineural invasion (PNI) in esophageal and esophagogastric junction (EGJ) carcinoma.* Methods*. A comprehensive literature search for relevant reports published up to July 2015 was performed using Pubmed and Embase databases. The pooled HR and 95% CI for overall survival (OS) and disease-free survival (DFS) were used to assess the prognostic value. The association of PNI with pathological characteristics was evaluated by OR and 95% CI.* Results*. A total of 13 cohorts were retrieved, covering 2770 patients treated by surgery. The cumulative analysis revealed a statistical correlation between PNI and poor OS (HR = 1.76, 95% CI: 1.54–2.20, and *P* < 0.00001), as well as poor DFS (HR = 1.96, 95% CI: 1.42–2.71, and *P* < 0.001). Moreover, analysis of 1475 patients showed improved PNI in T3 + T4 (OR = 0.39, 95% CI: 0.21–0.70, and *P* = 0.002), N+ (OR = 0.52, 95% CI: 0.40–0.69, and *P* < 0.00001), and G3 + G4 (OR = 0.66, 95% CI: 0.48–0.90, and *P* = 0.008) patients compared with T1 + T2, N−, and G1 + G2 ones, respectively. No significant heterogeneity was found between the studies.* Conclusions*. PNI is an adverse prognostic biomarker in esophageal and EGJ carcinoma. Moreover, PNI implies advanced T, N stage and poor cell differentiation.

## 1. Introduction

Esophageal carcinoma is the 6th most common cause of cancer death worldwide with very aggressive biological behavior [1]. Despite the development in diagnostic modalities and stratified treatment due to TNM classification in recent years, the prognosis of patients remains poor with an overall 5-year survival of 15%–25% [2]. Identification of novel prognostic factors may be helpful to attain more individualized and efficient cancer therapy.

Perineural invasion (PNI) is the process of neoplastic invasion of nerves which facilitates aggressive growth and spread of tumor cells [3]. In multiple malignancies such as head and neck cancer, pancreatic adenocarcinoma, colorectal cancer, gastric and prostate cancer, PNI occurrence is correlated with high recurrence rates, aggressive behavior, and poor survival [4–8]. Given the considerable clinical impact, PNI was added to the 7th UICC/AJCC TNM classification as a new parameter [9]. But the investigations on prognostic role of PNI in esophageal and esophagogastric junction (EGJ) carcinoma have released inconsistent results. Although recognized as independent prognostic factor in several studies [10, 11], PNI was reported by Ochiai et al. not significantly related to overall survival (OS) in esophageal cancer [12]. Moreover, PNI was found to be significant factor for OS in adenocarcinoma of esophagus only but not in squamous cell carcinoma (SCC) by univariate analysis, while multivariate analysis failed to draw the conclusion [13]. The aim of our study was to evaluate the prognostic value of PNI in esophageal and EGJ carcinoma by systematically reviewing the available evidence.

## 2. Methods

### 2.1. Search Strategy

We searched Pubmed and Embase for studies correlating the presence of PNI with patients' survival in esophageal and EGJ carcinoma published up to July 30, 2015. The search terms included “perineural invasion”, “neural invasion”, “esophageal carcinoma”, “esophagogastric junction carcinoma”, “gastric cardiac carcinoma”, and “prognosis”. The studies were limited to human articles published in English. In addition, citation lists of retrieved articles were manually screened to identify potentially relevant studies.

### 2.2. Selection Criteria

Studies were included if they met the following criteria: (1) the diagnoses of esophageal and EGJ carcinoma and PNI were based on pathological examination; (2) the studies reported the outcome of OS or disease-free survival (DFS); (3) they provided hazard ratio (HR) with confidence interval (CI) or original data sufficient for calculating them. Studies published as abstracts only were excluded.

### 2.3. Data Extraction and Quality Assessment

Data were extracted independently by two authors (Gao and Wang) using a standard protocol. The following information was collected from each study: first author, year of publication, study design, patients characteristics (country of origin, number, age, sex, duration of follow-up, PNI positive rate, etc.), clinicopathological features (perioperative treatment, TNM stage, pathological grade, cell type, etc.), and survival (OS and DFS), whose data was summarized by HR and its 95% CI. Quality assessment was performed for each eligible study using the Newcastle-Ottawa quality assessment scale (NOS) [14]. Studies with NOS scores ≥6 were considered to be of high quality.

### 2.4. Statistical Analysis

The meta-analysis was performed using Review Manager V5.3 software (Copenhagen, The Nordic Cochrane Centre, The Cochrane Collaboration). For studies whose HR and 95% CI were not reported directly, we estimated them according to the method of Tierney [25]. *Q* statistical test and *I* ^2^ value were used to assess the heterogeneity of these studies. *P* < 0.1 and/or *I* ^2^ > 50% was considered as significant heterogeneity and the cause was analyzed [26]. Pooled estimates of the HRs were obtained by fixed-effect model where no significant heterogeneity was found. Otherwise, a random-effect model was used. Publication bias was assessed using a funnel plot. Subgroup analyses were performed by ethnicity and cell type.

## 3. Results

### 3.1. Search Results and Study Characteristics

**Figure 1:**
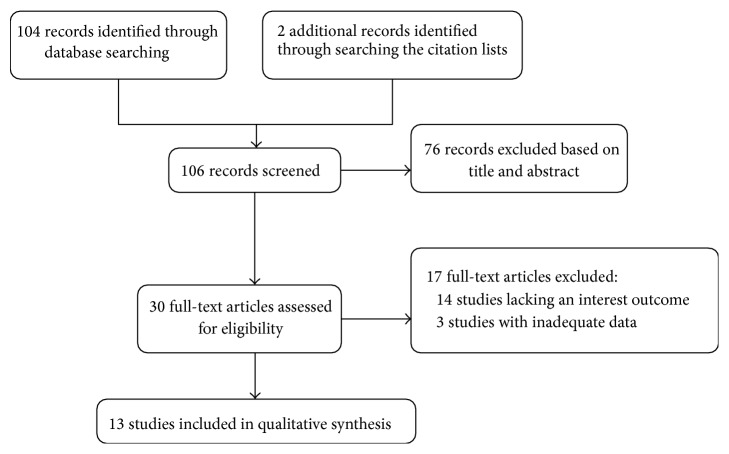
Flow diagram of study selection procedure.

The search strategy retrieved one hundred and four unique articles, of which 76 were excluded after the first screening of titles and abstracts. Hand searching of the citation lists identified two additional articles. After reviewing the full texts of the remaining 30 articles potential to be eligible, 17 studies were excluded for lacking an interest outcome or inadequate data, leaving 13 studies [10, 11, 13, 15–24] comprising 2770 patients treated by surgery with/without perioperative chemo(radio)therapy for final inclusion in the meta-analysis (Figure 1).

All the included studies were retrospective cohort studies. They were published between 1995 and 2015, with sample size ranging from 26 to 691 (median = 142). Six of these studies were based on Asian population [10, 11, 16, 19, 22, 24], 5 were European [13, 15, 18, 20, 21], and the other two were American [17, 23]. A median of PNI positive rate was 33.3% (5.5% to 61.3%). Three studies investigated patients with SCC [10, 11, 20], six with non-SCC [15, 16, 21–24], and the remaining 4 with mixed pathological types [13, 17, 18, 20]. Among the 13 cohorts, HRs and 95% CIs for OS were directly reported in nine. Four studies [10, 19, 20, 22] simultaneously reported DFS as the outcome in which two HRs were provided directly. For the remaining 4 [13, 16, 17, 21] and 2 [10, 22] studies on OS and DFS, HRs and 95% CIs were calculated using data provided in the original articles. Detailed clinical and pathological characteristics are summarized in Table 1.

### 3.2. OS and DFS Related to PNI Status

**Figure 2:**
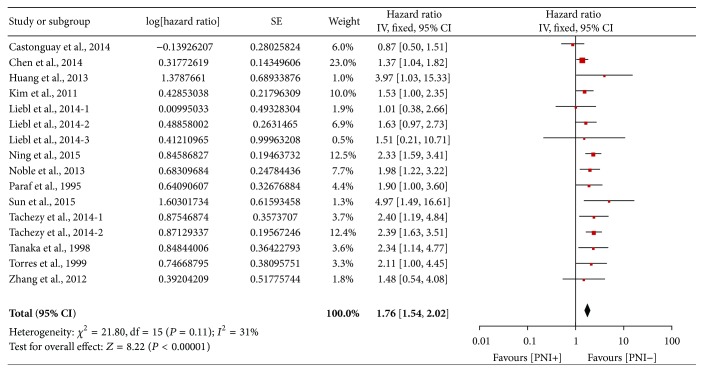
Forest plot of the combined hazard ratio (HR) for the association of PNI with OS.

**Figure 3:**
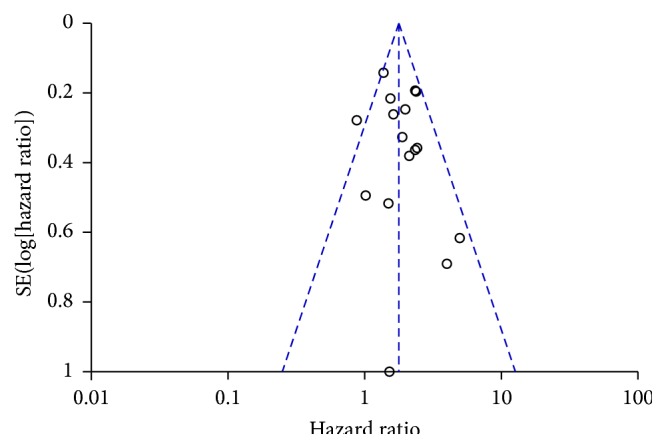
Funnel plot to visualize the potential publication bias in the prognostic assessment of PNI on OS.

A total of 16 HR values for OS were included because HRs of AEG (adenocarcinoma of esophagogastric junction) I, AEG II/III, SCC in Liebl et al.'s study [18], and HRs of SCC, adenocarcinoma in Tachezy et al.'s study [13], were all reported separately, which were labeled in our analysis as Liebl et al.'s, 2014-1, Liebl et al.'s, 2014-2, Liebl et al.'s, 2014-3, Tachezy et al.'s, 2014-1, and Tachezy et al.'s, 2014-2, respectively. A pooled analysis of 13 cohorts including 16 HRs demonstrated that PNI was associated with poor OS in esophageal and EGJ carcinoma (HR = 1.76, 95% CI: 1.54–2.20, and *P* < 0.00001; Figure 2). Because no significant heterogeneity was found (*P* = 0.11, *I* ^2^ = 31%), a fixed-effect model was used here. There was no evidence for publication bias (Figure 3).

**Figure 4:**
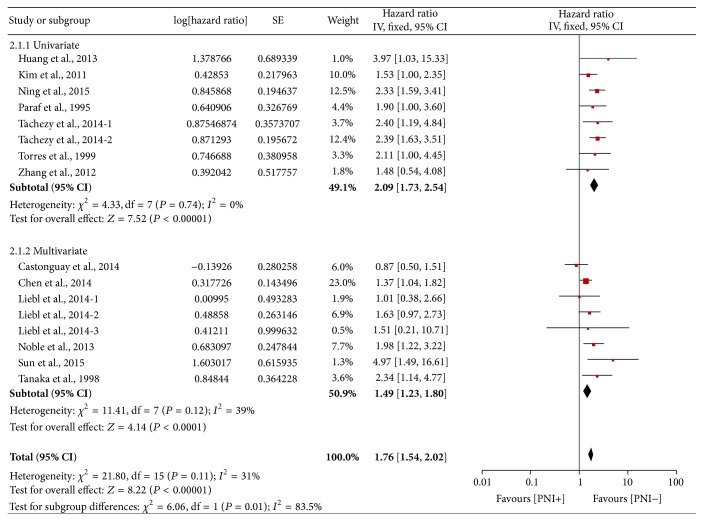
Forest plot of the combined HR for the association of PNI with OS in univariate and multivariate subgroups.

Among the 13 studies, seven [13, 16, 17, 19, 21, 23, 24] were investigated by univariate analysis with HRs ranging from 1.48 to 3.97, and six [10, 11, 15, 18, 20, 22] were by multivariate analysis with HRs ranging from 0.87 to 4.97. To further explore the study heterogeneity, we performed subgroup analysis by univariate and multivariate HRs. Again, PNI predicted poor OS in both subgroups (univariate analysis subgroup: HR = 2.09, 95% CI: 1.73–2.54, and *P* < 0.00001; multivariate analysis subgroup: HR = 1.49, 95% CI: 1.23–1.80, and *P* < 0.0001; Figure 4). There was no significant heterogeneity in either subgroup (*P* = 0.74 and 0.12, *I* ^2^ = 0 and 39%, resp.)

**Figure 5:**
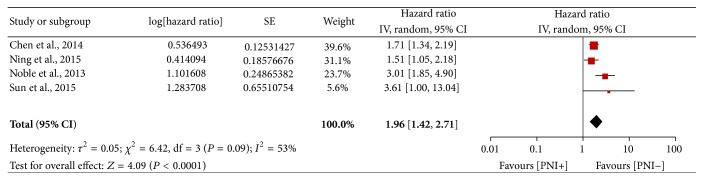
Forest plot of the combined HR for the association of PNI with DFS.

Only four studies [10, 19, 20, 22] comprising 948 patients reported DFS as the outcome simultaneously. A combined analysis revealed a random-effect HR of 1.96 (95% CI: 1.42–2.71, *P* < 0.0001; Figure 5), suggesting PNI is an unfavourable predictor of DFS. Moderate heterogeneity was observed in the comparison (*P* = 0.09, *I* ^2^ = 53%).

### 3.3. Stratified Analyses

**Figure 6:**
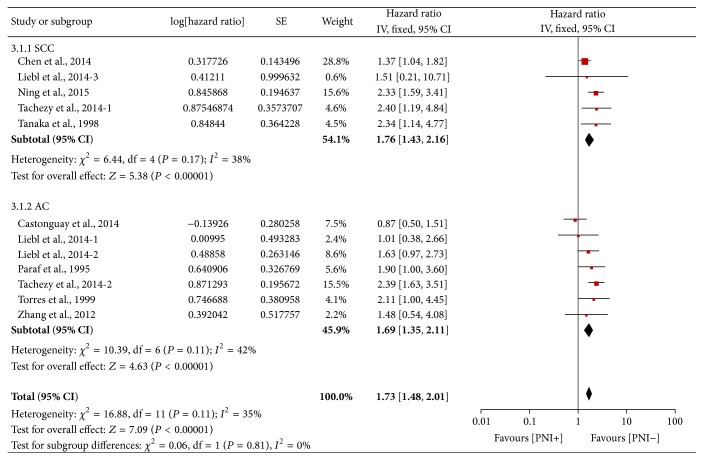
Forest plot of the combined HR for the association of PNI with OS in SCC and AC subgroups.

In order to identify the value of PNI on different pathological type of esophageal and EGJ carcinoma, we stratified 12 HRs whose cell type was reported as SCC or adenocarcinoma (AC) only. A total of 1183 patients were included in SCC subgroup and 1007 in AC subgroup. A pooled data showed that PNI was an unfavourable indicator of OS in SCC subgroup with HRs of 1.76 (95% CI: 1.43–2.16, *P* < 0.00001), as well as in AC subgroup with HR of 1.69 (95% CI: 1.35–2.11, *P* < 0.00001) (Figure 6). Mild heterogeneity was found in both subgroups (SCC subgroup: *P* = 0.17, *I* ^2^ = 38%; AC subgroup: *P* = 0.11, *I* ^2^ = 42%).

**Figure 7:**
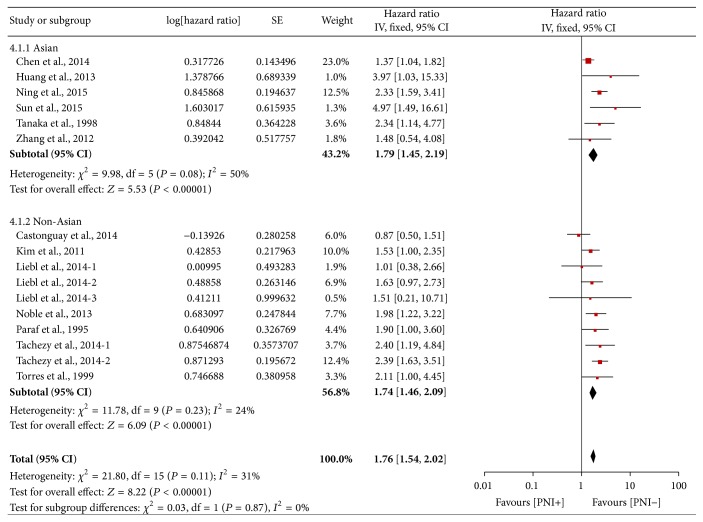
Forest plot of the combined HR for the association of PNI with OS in Asian and non-Asian subgroups.

The stratified analysis comparing the Asian and non-Asian subgroups yielded similar results. In Asian subgroup comprising 990 subjects, the pooled fixed HR was 1.79 (95% CI 1.45–2.19, *P* < 0.0001), while in non-Asian subgroup analysis of 1780 subjects yielded a pooled HR of 1.74 (95% CI: 1.46–2.09, *P* < 0.00001) (Figure 7), indicating that PNI is prognostically important in esophageal and EGJ carcinoma regardless of the geographic area.

### 3.4. Correlation between PNI and Pathological Characteristics

**Figure 8:**
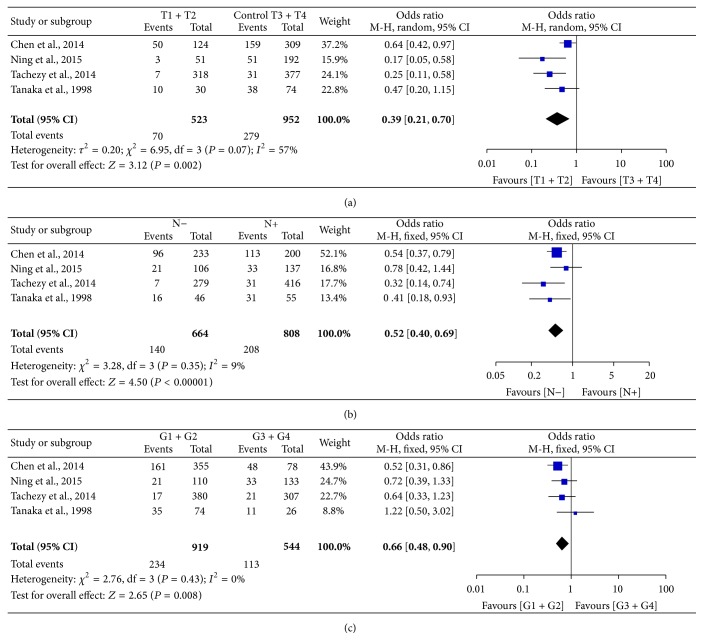
Forest plot of the pooled OR for the association of PNI with T stage (a), N stage (b), and histological grade (c).

Only four cohorts [10, 11, 13, 19] which included 1475 patients from China, Germany, and Japan reported PNI rate based on different T, N stages and histological grade (G). The combined analysis revealed improved PNI positivity in T3 + T4, N+, and G3 + G4 patients comparing with T1 + T2, N−, and G1 + G2 ones, with ORs of 0.39 (95% CI: 0.21–0.70, *P* = 0.002, Figure 8(a)), 0.52 (95% CI: 0.40–0.69, *P* < 0.0001, Figure 8(b)), and 0.66 (95% CI: 0.48–0.90, *P* = 0.008, Figure 8(c)), respectively, indicating presence of PNI was associated with advanced T, N stage and poor cell differentiation. While no significant heterogeneity was found in the comparison of different N and G status (*P* = 0.35 and 0.43, *I* ^2^ = 9% and 0%, resp.), moderate heterogeneity was found in the comparison of T stage (*P* = 0.07, *I* ^2^ = 57%).

## 4. Discussion

Although PNI is considered as a distinct route for dissemination and metastasis of tumor cells [27], there are conflicting reports on its prognostic significance in esophageal and EGJ carcinoma. Therefore, we performed the meta-analysis and demonstrated that PNI was an adverse factor of OS and DFS in patients treated with surgery. The pooled HR from multivariate analyses indicated that its prognostic effect on OS was independent of depth of invasion, lymph node status, and tumor grade as well as other clinicopathological features. In head and neck cancers, the great majority of patients with leptomeningeal carcinomatosis originating from PNI have no evidence of lymph node metastasis, confirming that the process of PNI is a distinct way of metastasis [28, 29]. We advocate that PNI might be included in the TNM staging system of esophageal and EGJ carcinoma as a new prognostic parameter, helping to identify patients who might benefit from additional treatment or intensified follow-up.

In the NCCN guideline for esophageal and EGJ cancers (version 3, 2015), PNI is recommended as a high-risk feature to guide the postoperative treatment of lower-esophagus and EGJ adenocarcinoma patients. However, our stratified analysis by cell type showed that positivity of PNI predicted poor OS in both SCC and AC subgroups, which would probably extend its importance in clinical practice.

Asia, often referred to as “esophageal cancer belt,” is a high-risk area with distinct epidemiological and histological properties from non-Asian area [1]. However, our analysis based on Asian and non-Asian population did not affect the prognostic value of PNI, suggesting the predictive significance is independent of ethnicity.

We also evaluated the relationship of PNI with the well-known and established prognostic criteria, including T, N stage and histological grade, which constitute the basis for the AJCC TNM classification of esophageal and EGJ cancer [30]. As indicated by the pooled analyses, PNI is statistically significantly related to infiltration depth (described as T stage) and lymph node metastasis, suggesting that PNI is an important biological marker of tumor invasion. Furthermore, improved PNI positivity in poorly differentiated tumors implied that PNI indicated more malignant behavior of tumor cells. The underlying mechanism may be explained by the presence of cross talk between tumor cells and nerve terminations around, which is supported by substantial experimental researches [31–33]. In this biological process, neurotransmitters and neuropeptides secreted by nerve terminations act as molecule determinants and promote tumor invasion and metastasis [27, 34].

This meta-analysis, to our knowledge, is the first study to systematically evaluate the prognostic significance of PNI in esophageal and EGJ carcinoma. Only mild to moderate heterogeneity was detected between these studies, which can be attributed mainly to inconsistent definitions and detection methods of PNI, different geographic areas, editions of TNM classification, tumor sites, histological types, and therapy strategies. Notably, the included cohorts were all retrospective researches which may cause heterogeneity themselves. In spite of this discrepancy, the prognostic significance of PNI was not affected. Standardized definition and detection method are desirable for getting accurate PNI positive rate in the future.

In conclusion, PNI is an independent and adverse prognostic indicator which implies aggressive and metastatic behaviors of tumor cells in esophageal and EGJ carcinoma patients treated with surgery. Introduction of PNI as a novel prognostic parameter may provide additional information to guide clinical therapy and follow-up. Furthermore, prospective cohort studies with large samples are expected to validate our findings in the future.

## Figures and Tables

**Table 1:** Baseline characteristics of included cohorts.

Study	Country/area	Number (male/female)	Tumor sites	PNI positive *n* (positive rate)	nCRT^1^/nCT^2^ number	TNM edition	Stage of TNM	Outcome	SQ^3^
Castonguay et al., 2014 [15]	Canada	103 (86/17)	Esophagus	57 (55.3%)	5	AJCC/UICC 7th	I–IV	OS^4^	7
Chen et al., 2014 [10]	China	433 (321/112)	Esophagus	209 (48.3%)	0	AJCC/UICC 7th	I–III	DFS^5^, OS	7
Huang et al., 2013 [16]	China	42 (33/9)	Esophagus	14 (33.3%)	0	AJCC/UICC 7th	I–IV	OS	5
Kim et al., 2011 [17]	America	266 (196/70)	Esophagus and EGJ^6^	29 (10.9%)	162	NR^7^	NR	OS	6
Liebl et al., 2014 [18]	Germany	311 (249/62)	Esophagus and EGJ	132 (42.4%)	0	AJCC/UICC 7th	I–IV	OS	8
Ning et al., 2015 [19]	China	243 (194/49)	Esophagus	54 (22.2%)	NR	AJCC/UICC 7th	II-III	DFS, OS	7
Noble et al., 2013 [20]	UK	246 (195/51)	Esophagus and EGJ	34 (14.0%)	151	AJCC/UICC 7th	I–IV	DFS, OS	7
Paraf et al., 1995 [21]	France	67 (61/6)	Lower esophagus	26 (38.2%)	0	NR	NR	OS	5
Sun et al., 2015 [22]	Taiwan	26 (23/3)	Esophagus and EGJ	5 (19.2%)	0	AJCC/UICC 7th	I–III	DFS, OS	6
Tachezy et al., 2014 [13]	Germany	695 (555/140)	Esophagus	38 (5.0%)	52	AJCC/UICC 7th	I–IV	OS	7
Tanaka et al.,1998 [11]	Japan	104 (84/20)	Esophagus	48 (46.2%)	22	AJCC/UICC 1993	NR	OS	7
Torres et al., 1999 [23]	America	96 (83/13)	Lower esophagus	31 (32.3%)	61	AJCC/UICC 1993	I–IV	OS	5
Zhang et al., 2012 [24]	China	142 (109/33)	EGJ II-III	87 (61.3%)	0	AJCC/UICC 7th	I–IV	OS	6

^1^nCRT: neoadjuvant chemoradiotherapy; ^2^nCT: neoadjuvant chemotherapy; ^3^SQ: study quality based on the Newcastle-Ottawa scale; ^4^OS: overall survival; ^ 5^DFS: disease-free survival; ^6^EGJ: esophagogastric junction; ^7^NR: not reported.
